# E-Cadherin Can Replace N-Cadherin during Secretory-Stage Enamel Development

**DOI:** 10.1371/journal.pone.0102153

**Published:** 2014-07-11

**Authors:** Xiaomu Guan, Felicitas B. Bidlack, Nicole Stokes, John D. Bartlett

**Affiliations:** 1 Department of Mineralized Tissue Biology and Harvard School of Dental Medicine, The Forsyth Institute, Cambridge, Massachusetts, United States of America; 2 Laboratory of Mammalian Cell Biology and Development, Howard Hughes Medical Institute, The Rockefeller University, New York, New York, United States of America; University of Colorado, Boulder, United States of America

## Abstract

**Background:**

N-cadherin is a cell-cell adhesion molecule and deletion of N-cadherin in mice is embryonic lethal. During the secretory stage of enamel development, E-cadherin is down-regulated and N-cadherin is specifically up-regulated in ameloblasts when groups of ameloblasts slide by one another to form the rodent decussating enamel rod pattern. Since N-cadherin promotes cell migration, we asked if N-cadherin is essential for ameloblast cell movement during enamel development.

**Methodology/Principal Findings:**

The enamel organ, including its ameloblasts, is an epithelial tissue and for this study a mouse strain with N-cadherin ablated from epithelium was generated. Enamel from wild-type (WT) and N-cadherin conditional knockout (cKO) mice was analyzed. μCT and scanning electron microscopy showed that thickness, surface structure, and prism pattern of the cKO enamel looked identical to WT. No significant difference in hardness was observed between WT and cKO enamel. Interestingly, immunohistochemistry revealed the WT and N-cadherin cKO secretory stage ameloblasts expressed approximately equal amounts of total cadherins. Strikingly, E-cadherin was not normally down-regulated during the secretory stage in the cKO mice suggesting that E-cadherin can compensate for the loss of N-cadherin. Previously it was demonstrated that bone morphogenetic protein-2 (BMP2) induces E- and N-cadherin expression in human calvaria osteoblasts and we show that the N-cadherin cKO enamel organ expressed significantly more BMP2 and significantly less of the BMP antagonist Noggin than did WT enamel organ.

**Conclusions/Significance:**

The E- to N-cadherin switch at the secretory stage is not essential for enamel development or for forming the decussating enamel rod pattern. E-cadherin can substitute for N-cadherin during these developmental processes. *Bmp2* expression may compensate for the loss of N-cadherin by inducing or maintaining E-cadherin expression when E-cadherin is normally down-regulated. Notably, this is the first demonstration of a natural endogenous increase in E-cadherin expression due to N-cadherin ablation in a healthy developing tissue.

## Introduction

Cadherins are a class of transmembrane proteins and are the major adhesion molecules located within adherens junctions. They can mediate cell-cell adhesion through their extracellular domain and their cytosolic domains connect to the actin cytoskeleton by binding to catenins [1]. The presence of cadherins is absolutely essential for enamel formation because ablation of the molecule (p120-catenin) responsible for stabilizing cadherins to the cell surface results in severely malformed dental enamel in mice [2].

Among the more than 20 members of the classical cadherin family, N-cadherin (cadherin-2) is of interest due to its essential role in several developmental processes [3]. It is one of only three cadherins, including E-cadherin and VE-cadherin that are essential for embryonic development [4], [5], [6], [7]. Although global N-cadherin knockout embryos can form neural tubes, somites and myocardium, these tissues are defective and lead to early embryonic lethality [4]. To perturb N-cadherin function in specific cellular contexts, genetic loss-of-function approaches have been utilized. For example, N-cadherin ablation in mouse myocardium leads to dissolution of the intercalated disc structure and remodeling of gap junctions. This highlights its essential role in mediating cardiomyocyte adhesion and thus maintaining heart structural integrity [8], [9], [10]. In eye development, conditional deletion of N-cadherin in embryonic lens results in microphthalmia and lens epithelial cell deterioration [11]. Although N-cadherin is essential for several development processes, overlapping function among different cadherin family members does exist. For example, cardiac-specific expression of an E-cadherin transgene in an N-cadherin-deleted embryo can rescue the cardiac adhesion defect [5]. Also, during intestinal development, transgenic N-cadherin can structurally substitute for E-cadherin to establish organ architecture [12]. These results suggest that E- and N-cadherin function can be interchangeable in certain cellular contexts.

Enamel development progresses through defined stages [13]. During the secretory stage, groups of ameloblasts slide by one another to form the characteristic rodent decussating enamel prism pattern [14], [15], [16]. Therefore, cell movement and communication play important roles in enamel development, but the detailed mechanism is not well characterized. Previously, we identified a cadherin switch in ameloblasts during the secretory stage where E-cadherin was down-regulated while N-cadherin was significantly up-regulated [17]. An E- to N-cadherin switch is associated with epithelial-mesenchymal transition (EMT) during cancer invasion where epithelial cells become motile [18]. Specifically, in breast cancer cells, elevated N-cadherin levels lead to increased cell mobility and metastasis [19], [20]. In addition, N-cadherin promotes directional chain migration of cerebellar granule neurons by regulating cell-cell contacts through the remodeling of adherens junctions [21]. These data indicate that N-cadherin plays important cell adhesion roles in development, disease and cell motility.

In this study we hypothesize that N-cadherin promotes ameloblast mobility during the secretory stage of enamel development. Given that deletion of N-cadherin is embryonic lethal, conditional epithelial N-cadherin knockout mice (cKO) were generated using K14-*Cre/LoxP* recombination technology [22] where the keratin-14 promoter directed *Cre* expression in epithelial tissues including the enamel organ and its ameloblasts [2]. The cKO mice were viable and the physical properties of their enamel (prism pattern, hardness, relative mineral content) were examined. In addition, ameloblasts were assessed for cadherin protein expression and enamel organs were assessed for cadherin and cadherin associated signaling molecule expression levels.

## Materials and Methods

### Ethics Statement

All animals used in this study were housed in an Association for Assessment and Accreditation of Laboratory Animal Care (AAALAC) accredited facilities (animal welfare assurance number: A3051-01) and were treated humanely based on a protocol approved by the Institutional Animal Care and Use Committee (IACUC) at The Forsyth Institute. Experimental protocols were designed along institutional and National Institutes of Health guidelines for the humane use of animals.

### Generation of N-Cadherin cKO Mice

Mice with *loxP* sites encompassing exon 1 of the N-cadherin gene were purchased from Jackson Laboratory (B6.129S6(SJL)-*Cdh2^tm1Glr^*/J). Mice with transgenic *Cre* expressed by the keratin 14 promoter (Tg(KRT14-cre)1Efu) were a generous gift from Dr. Elaine Fuchs at Rockefeller University. There were two steps involved in the breeding scheme. First, the K14-Cre mice were bred with the N-cadherin (N-cad)-*LoxP*+/+ mice. The K14-*Cre*-N-cad-*LoxP*+/− offspring (∼50%) were selected and further bred with N-cad-*LoxP*+/+ mice. In this second step, the offspring with the K14-*Cre*-N-cad-*LoxP*+/+ genotype (∼25%) were selected and their enamel was characterized.

### Quantitative Real-time PCR (qPCR)

mRNA was extracted from first molar enamel organs of 5 day-old WT and N-cadherin cKO mice, and was reverse transcribed to cDNA for qPCR analysis. qPCR reactions were performed on a Roche LightCycler 480 with each reaction containing LightCycler 480 SYBR Green I Master (Roche, Basel, Switzerland), cDNA, and 0.25 uM forward and reverse primers. The primers used in this study are listed in Table 1. The instrument was programmed as follows: 3 min at 95°C for initial denaturation, and 95°C 15 sec, 58°C 15 sec, 72°C 15 sec for 40 cycles, followed by a melting curve. Standard curves were generated with each primer set using control samples prepared by pooling all cDNA samples together and making a 4-fold dilution series covering all sample concentrations. Reaction efficiencies and gene expression levels were calculated as previously described [23]. All expression levels were normalized to the internal reference control gene *Rn18s* (18S ribosomal RNA) given its high and stable expression level, and were presented as relative ratios to the WT day-5 data.

**Table 1:** Primers used for quantitative real-time PCR (qPCR).

Gene	5′ Primer	3′ Primer
Cdh2 (N-cad)	TGAAACGGCGGGATAAAGAG	GGCTCCACAGTATCTGGTTG
Cdh1 (E-cad)	GGTTTTCTACAGCATCACCG	GCTTCCCCATTTGATGACAC
Cdh3 (P-cad)	GCACCATGCAGACAATGG	AATATTGGTGGCATCACCCAC
Cdh5 (VE-cad)	CTTCCAGCGACACTTCTACC	TTCCCTGCTTGGTTATTCGG
Cdh11	CAATATCGTTGATGGAGACGGC	ACATTGGCGGCCTCTATCTT
p120	GGGAAAAAGCCTACAGAGGAT	ACAAGTTCTGGATAGCTCCG
β-catenin	ATTGATTCGAAACCTTGCCC	AGCTCCAGTACACCCTTCTA
Bmp2	TAGATCTGTACCGCAGGCA	CCGTTTTCCCACTCATCTCT
Nog	CGGCCAGCACTATCTACACA	GCGTCTCGTTCAGATCCTTC
Rn18s	GTAACCCGTTGAACCCCATT	CCATCCAATCGGTAGTAGCG

### MicroComputed Tomography (μCT) Scanning

Soft tissues were removed from adult WT and N-cadherin cKO mouse hemi-mandibles. The hemi-mandibles including the mandibular incisors were then washed with 70% EtOH, and the degree of enamel mineralization was assessed using a Scanco μCT 40 (Scanco Medical, Wayne PA, USA). The samples were scanned in 70% EtOH with settings of 70 kV, 114 mA, and 0.01 mm isotropic voxels. 3D images of enamel layers were reconstructed with μCT 40 evaluation software. Images were generated by using the same threshold value for the WT and N-cadherin cKO samples.

### Scanning Electron microscopy (SEM)

Hemi-mandibles from adult WT and N-cadherin cKO mice were washed, dehydrated with graded ethanol solutions, and air-dried. Mandibular incisors were dissected from the hemi-mandibles, and the erupted tooth portion was fractured perpendicular to the growth axis and close to the alveolar bone, mounted onto metallic stubs, and sputter-coated with gold. The samples were imaged using a Zeiss EVO LS10 scanning electron microscope (Carl Zeiss AG, Jena, Germany) under high vacuum with 7.0–9.5 mm working distance, 5–15 kV, and 3–8 pA. Analyses were performed with 3 mice per genotype.

### Vickers Microhardness Testing

Maxillary incisors from adult WT and N-cadherin cKO mice were washed and dehydreated with graded ethanol. Incisors were embedded sagittally in epoxy resin (EMS, Hatfield, PA), and polished in a circling motion to 0.25 µm with a series of diamond suspensions (EMS, Hatfield, PA). Enamel microhardness was measured by a Leco M 400 HI testing machine (Leco, St. Joseph, MI) with a Vickers tip and a load of 25 g for 5 sec. Vickers microhardness was calculated by ConfiDent hardness measuring system (Leco, St. Joseph, MI) and the results were averaged from ten indentations per sample and 4 mice per genotype.

### Immunohistochemistry

WT and N-cadherin cKO mouse incisor sections were deparaffinized and rehydrated. Sections stained for N-cadherin were subjected to microwave antigen retrieval in 10 mM citrate buffer for 10 min, blocked with 1.5% goat serum in phosphate-buffered saline (PBS) for 30 min at room temperature and incubated with rabbit polyclonal N-cadherin antibody (Pierce, Rockford, IL) at a 1∶1000 dilution overnight at 4°C. Sections stained for Pan-cadherin were also subjected to microwave antigen retrieval in 10 mM citrate buffer for 10 min, blocked with 1.5% goat serum in PBS for 30 min at room temperature and incubated with rabbit Pan-cadherin antibody (Cell Signaling Technology, Danvers, MA) at a 1∶500 dilution overnight at 4°C. Finally, sections stained for E-cadherin were incubated in M.O.M mouse Ig blocking reagent (10% in PBS) overnight at 4°C, followed by 1 h incubation with 1∶250 diluted mouse monoclonal E-cadherin antibody (BD Biosciences, Franklin Lakes, New Jersey). Sections without primary antibody were used as negative controls. Endogenous peroxidase activity was quenched with 1% H_2_O_2_ in methanol for 30 min before primary antibody incubation. Antibody binding was visualized using the Vector M.O.M Immunodetection Kit for E-cadherin and the Vectastain Elite ABC Kit for N-cadherin and Pan-cadherin with ImmPACT DAB peroxidase substrate (Vector Laboratories, Burlingame, CA). Sections were counterstained with 0.1% Fast Green in PBS for 2 min, dehydrated, and mounted.

### Statistics

Unpaired t-tests were performed using Microsoft Excel version 14.3.0 (Microsoft Corporation, Redmond, WA) to analyze the significance of qPCR result differences.

## Results

### N-cadherin Expression was Disrupted in cKO Mouse Enamel Organs

**Figure 1:**
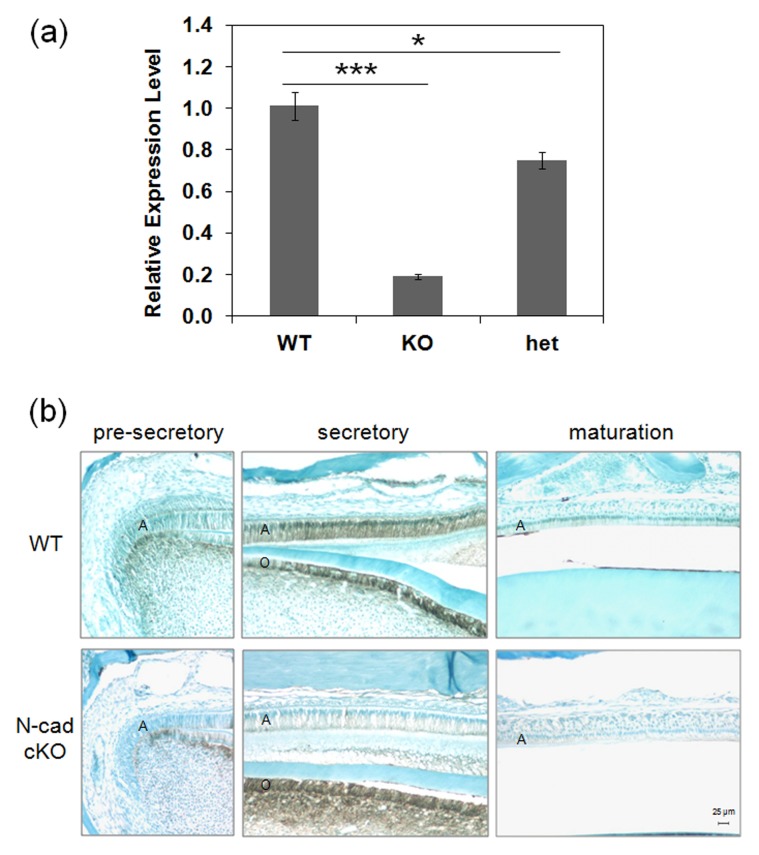
(**a**) qPCR analysis of N-cadherin gene expression in wild-type (WT) and N-cadherin conditional knockout (cKO) mouse enamel organs. mRNA was extracted from postnatal day-5 enamel organs from 3 mice per genotype for qPCR analysis. Results are presented as expression ratios relative to the WT levels. The ablated N-cadherin cKO enamel organs (K14-*Cre*-N-cadherin-*LoxP*+/+) had 0.19 fold of the WT N-cadherin expression level (***, p<0.001) and the heterozygous ablated mice (K14-*Cre*-N-cadherin-*LoxP*+/−) had 0.75 fold of the WT expression level (*, p<0.05). (**b**) N-cadherin protein expression was ablated in the ameloblast layer of K14-*Cre*-N-cadherin-*LoxP*+/+ mice. Immunohistochemical staining of N-cadherin was performed on paraffin-imbedded incisor sections from both WT and N-cadherin cKO mice. In WT mice N-cadherin was not expressed highly in pre-secretory stage ameloblasts, but was strongly up-regulated during the secretory stage and was later down-regulated when the ameloblasts progressed into the maturation stage. In contrast, regardless of developmental stage, N-cadherin expression was not observed in the N-cadherin cKO ameloblasts demonstrating that N-cadherin expression was successfully deleted in these mice. A, ameloblast layer; O, odontoblast layer.

To evaluate N-cadherin expression level from N-cadherin cKO and WT mice, qPCR was performed on WT, K14-*Cre*-N-cad-*LoxP*+/−, and K14-*Cre*-N-cad-*LoxP*+/+ enamel organs. Our previous results demonstrated that N-cadherin was significantly up-regulated in secretory stage ameloblasts [17]. So, RNA from postnatal day 5 (secretory stage) enamel organs was extracted and assessed for N-cadherin expression level. Mice with N-cadherin expression ablated from both alleles had less than 20% of the expression observed in WT mice (Fig. 1a). The heterozygous deletion had approximately three quarters of the WT expression level.

Immunohistochemistry was also performed on WT and N-cadherin cKO incisor sections to confirm N-cadherin ablation in the ameloblasts of the enamel organ (Fig. 1b). N-cadherin was present at low levels in the WT pre-secretory stage ameloblasts, was strongly up-regulated when enamel development progressed to the secretory stage, and was later down-regulated in the maturation stage of enamel development. In contrast, N-cadherin was not observed in the cKO ameloblasts starting from the pre-secretory stage through the maturation stage of development. Therefore, immunohistochemical results demonstrated that in N-cadherin cKO mice, N-cadherin expression was ablated in ameloblasts of the enamel organ. In addition, no obvious difference in ameloblast morphology between WT and cKO samples was observed (Fig. 1b).

### µCT and SEM Characterization of N-cadherin cKO Enamel

**Figure 2:**
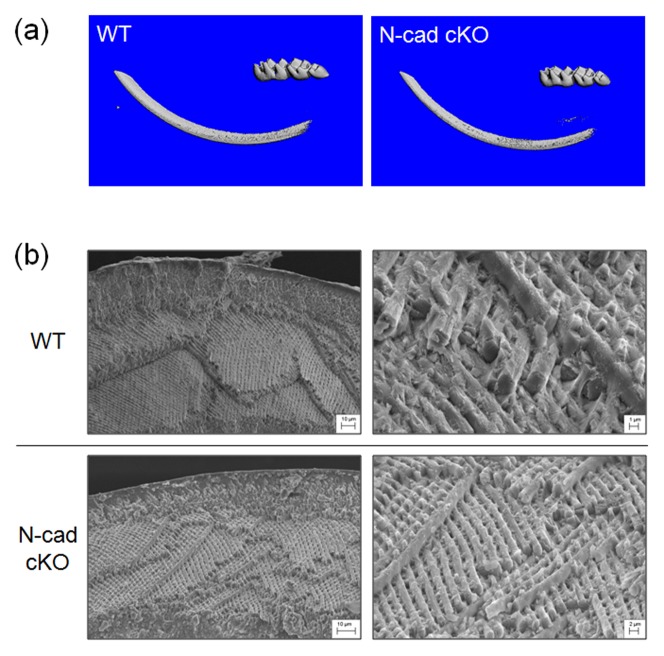
(**a**) N-cadherin cKO mice have normal enamel mineral levels. WT and N-cadherin cKO hemi-mandibles were subjected to micro-computed tomography (μCT). The same arbitrary threshold was selected for both genotypes and enamel 3D structures were generated. Results were repeated with 5 mice per genotype. (**b**) Enamel defects were not observed in the N-cadherin cKO mice. Scanning electron microscopy (SEM) analysis was performed on WT and N-cadherin cKO incisors. The cKO incisors had a smooth enamel surface without abnormalities. Fractured incisors revealed that both WT and cKO samples had similar enamel thicknesses and both had the characteristic decussating prism pattern. With higher magnification (right side) normal enamel rods were clearly observed for both samples.

To examine the physical properties of the cKO enamel, we first performed μCT scanning to quantify the degree of enamel mineralization. The same bone intensity threshold value was set for WT and N-cadherin cKO samples when reconstructing the 3D enamel images. Surprisingly, the 3D images (Fig. 2a) showed that no significant difference in enamel mineral density existed between the enamel from the WT and cKO mice. Both the N-cadherin ablated incisors and molars had normal amounts of mineralization.

We next performed scanning electron microscopy (SEM) analyses to assess enamel surface structure, thickness and prism pattern in WT and cKO mice. As for the lack of a difference in enamel mineral density, the absence of ameloblast N-cadherin expression also did not appear to affect enamel surface structure or enamel thickness. The incisors were also fractured and examined for the characteristic rodent enamel decussating rod pattern. Strikingly, the enamel rod pattern was completely normal in the N-cadherin cKO mice. In both WT and cKO samples, the decussating rod pattern was clearly present (Fig. 2b).

### Vickers Hardness Measurements

**Figure 3:**
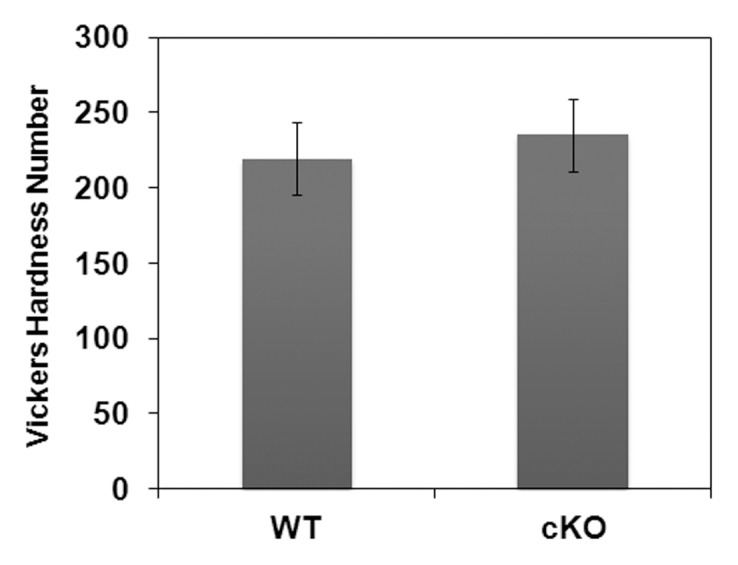
No difference in Enamel hardness between WT and N-cadherin cKO mice. Adult mouse incisors were harvested and indented for Vickers microhardness measurements. Enamel hardness from 4 mice per genotype was measured and results were averaged. WT samples have an averaged Vickers hardness number of 219.6±24.5, while N-cadherin cKO samples possess a slightly higher value of 235.1±23.8.

To assess if N-cadherin ablation results in softer-than-normal enamel, Vickers micro-hardness measurements were performed on enamel from WT and N-cadherin cKO mice. Four mice from each genotype were analyzed and the results were averaged. Enamel hardness did not change significantly with the loss of N-cadherin (Fig. 3). This result also suggests that N-cadherin is not essential for healthy enamel development and does not significantly affect the mineralization process.

### qPCR Analysis of Cell Adhesion Molecules

**Figure 4:**
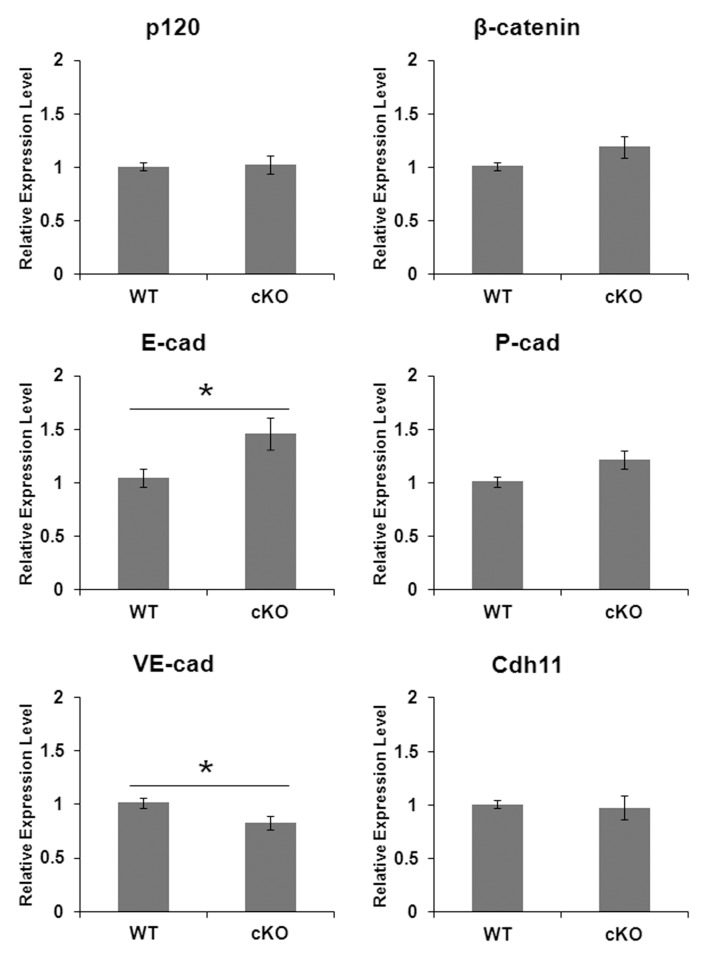
Gene expression analysis in WT and N-cadherin cKO mouse enamel organs. qPCR was performed on WT and N-cadherin cKO postnatal day-5 enamel organs with 7 mice assessed per genotype. No significant difference was observed in expression levels of p120 and β-catenin between WT and N-cadherin ablated enamel organs. Various cadherins were assessed for expression in secretory stage enamel organ and for those that were expressed, expression levels were assessed. A comparison of expression levels between WT and N-cadherin cKO enamel organs revealed that VE-cadherin expression was slightly but significantly reduced compared to WT and that E-cadherin expression was significantly increased by approximately 1.5 fold compared to WT. No significant differences by genotype were observed for P-cadherin or cadherin-11 (*, p<0.05).

Adherens junctions also contain signaling molecules that function to prevent cadherin internalization (p120-catenin; p120) and that are part of the complex that links cadherins to the actin cytoskeleton (β-catenin) [24]. We therefore sought to determine if these potential signaling molecules had altered expression levels in the absence of N-cadherin. However, no significant difference in p120 or β-catenin expression level was observed in secretory stage enamel organs from WT or N-cadherin cKO mice (Fig. 4). These data were important because they suggest p120 and β-catenin signaling pathways are not altered in the cKO mice. But, more importantly, since these adherens junction proteins were expressed at the same level in WT and cKO enamel organs, it suggests that even though N-cadherin was ablated from the cKO mice, a similar number of adherens junctions were present in the cKO and WT enamel organs. We therefore asked if other cadherins were up-regulated to compensate for the loss of N-cadherin.

To this end, mRNA from secretory stage enamel organs was extracted and gene expression of several cadherins was screened by qPCR. In addition to N-cadherin, we show that the enamel organ also expresses a comparable level of E-cadherin, P-cadherin, VE-cadherin and cadherin-11 (Fig. 4). Among the cadherins assayed, P-cadherin and cadherin-11 expression were not significantly different between WT and cKO secretory stage enamel organs. In contrast, relative to WT, VE-cadherin expression was slightly decreased (∼20%) whereas E-cadherin expression increased by approximately 50% in the cKO enamel organ (Fig. 4).

### E-cadherin Replaces N-cadherin in cKO Secretory Stage Ameloblasts

**Figure 5:**
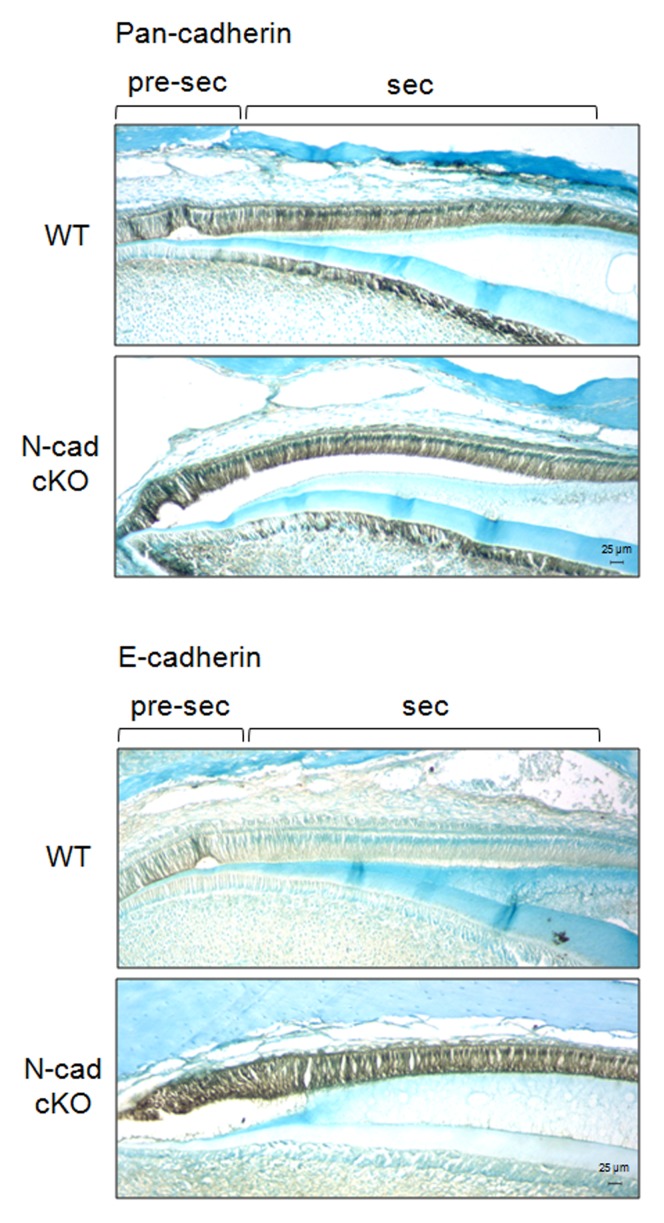
E-cadherin expression is abnormally elevated in secretory stage N-cadherin cKO ameloblasts. Immunohistochemical staining with pan-cadherin (total cadherin) or E-cadherin antibodies was performed on sectioned incisors from WT and N-cadherin cKO mice. For the pan-cadherin antibody, both genotypes stained pre-secretory and secretory stage ameloblasts with no apparent difference in staining intensity observed between WT and N-cadherin cKO ameloblasts. This indicates that total cadherin levels were similar between genotypes. E-cadherin stained well in the WT pre-secretory stage ameloblasts and faded as the ameloblasts entered the secretory stage. In contrast, E-cadherin staining in the N-cadherin cKO sample remained strong from the pre-secretory stage through the secretory stage.

We performed immunohistochemical procedures on WT and N-cadherin cKO incisor sections to confirm that increased E-cadherin transcripts in N-cadherin cKO enamel organ result in increased E-cadherin protein levels in ameloblasts. The sections were first stained with pan-cadherin antibody to assess the total amount of cadherins present in secretory stage enamel organ. As demonstrated in Figure 5, ameloblasts from both WT and N-cadherin cKO mice stained at approximately the same level from the pre-secretory stage to secretory stage, indicating that the total amount of cadherins was similar between these two genotypes. This was consistent with the p120 and β-catenin qPCR data suggesting that the total cadherin level did not change between WT and N-cadherin cKO mice.

E-cadherin immunohistochemistry was also performed and a striking difference in staining was observed between the WT and cKO sections. In WT mice E-cadherin was expressed in pre-secretory ameloblasts and was significantly down-regulated when enamel development progressed to the secretory stage. However, when N-cadherin was ablated, E-cadherin expression was enhanced and remained elevated throughout the secretory stage. Therefore, E-cadherin likely compensated for the loss of N-cadherin in the N-cadherin ablated ameloblasts.

### Identification of a Potential Mechanism by which E-cadherin Expression is Maintained During the Secretory Stage in N-cadherin cKO Mice

**Figure 6:**
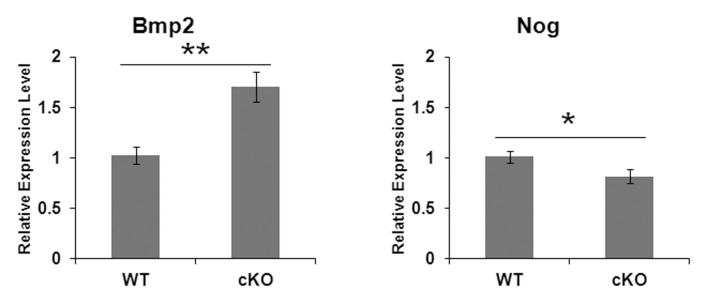
BMP2 signaling may be responsible for maintaining E-cadherin expression during the secretory stage in N-cadherin cKO enamel organs. qPCR was performed on WT and N-cadherin cKO postnatal day-5 enamel organs. *Bmp2* expression increased approximately 1.7 fold over WT levels in the N-cadherin ablated enamel organs (**, p<0.005). Conversely, the BMP signaling antagonist *Nog* decreased by approximately 80% of the WT expression level (*, p<0.05). Results were obtained from 4 mice per genotype.

We sought to identify a possible mechanism by which E-cadherin expression was maintained during the secretory stage in the cKO mice when E-cadherin is normally down-regulated. Previously, bone morphogenetic protein-2 (BMP2) was demonstrated to increase the expression of both E- and N-cadherin in human calvaria osteoblast cells [25]. We therefore quantified *Bmp2* expression levels by qPCR analysis in WT and cKO secretory stage enamel organs. We found that *Bmp2* was expressed at significantly increased levels in the N-cadherin cKO samples when compared to the WT samples (Fig. 6) indicating that BMP2 may play a role in maintaining E-cadherin expression in the cKO secretory stage enamel organ. Given that Noggin (NOG), an important signaling molecule in embryonic development, is a BMP signaling antagonist [26], we also quantified its expression levels. Relative to WT secretory stage enamel organs, *Nog* mRNA levels decreased approximately 20% in the N-cadherin cKO mice (Fig. 6). Therefore, both BMP2 and NOG are implicated in a possible mechanism by which E-cadherin maintains its expression during the secretory stage in the N-cadherin cKO secretory stage enamel organ.

## Discussion

Global knockout of N-cadherin in mice is embryonic lethal with severe cardiovascular defects [4], [5] and mice without N-cadherin expression in embryonic myocardium, endothelium, or neural crest cells are all embryonic lethal [27], [28], [29]. Mice with N-cadherin ablated from lens, osteoblasts or pancreas are viable, but have modest to severe defects during development [11], [30], [31]. This variety of phenotypes in N-cadherin ablated tissues illustrates the various functions of N-cadherin which are dependent on cellular context. During enamel development, N-cadherin is specifically up-regulated in secretory stage ameloblasts when cells slide by one another to form the decussating enamel rod pattern, suggesting that N-cadherin is involved in ameloblast adhesion and movement. Therefore, we specifically ablated N-cadherin expression in epithelial tissues which eliminates N-cadherin expression in the enamel organ and its ameloblasts responsible for enamel formation. The mice were viable and fertile without any apparent defect. We confirmed loss of N-cadherin gene expression in enamel organ by qPCR and immunohistochemistry. μCT, SEM, Vickers microhardness, and immunohistochemistry were performed to evaluate enamel physical properties. However, ablation of N-cadherin in dental epithelium resulted in no obvious defects in mouse enamel. Since N-cadherin, but not E-cadherin is associated with cell movement, we expected ameloblast movement to be restricted in the cKO mice which would result in an altered prism pattern. However, this did not occur. Therefore, it is likely that groups of ameloblasts were still able to slide by one another in the absence of N-cadherin.

This led us to screen various cadherins for possible expression within the secretory stage enamel organ to determine if another cadherin was substituting for N-cadherin in the ablated mice. Importantly, we identified two cadherins (VE-cadherin, cadherin-11) that had not previously been identified in the enamel organ. Expression level analysis revealed that E-cadherin expression was significantly higher than normal in the N-cadherin ablated secretory stage enamel organ and immunohistochemistry confirmed that the ameloblasts expressed substantially greater levels than normal of E-cadherin. This is a striking result because it is the first demonstration of a natural endogenous increase in E-cadherin expression due to N-cadherin ablation in a healthy developing tissue.

E-cadherin may compensate for the loss of N-cadherin because these cadherins are structurally similar. They both contain five extracellular cadherin domains with 46% conserved sequence and a cytosolic domain with 62% conserved amino acid sequence [32]. This similarity in structure may allow these cadherins to perform similar adhesion functions, while the differences in sequence may allow for interactions with cadherin-specific growth factor receptors for cell-context-dependent functions. Several studies have manipulated E- and N-cadherin expression *in vivo*, shedding light onto their interchangeability in mammalian developmental biology. Luo and coworkers showed that transgenic E-cadherin can functionally substitute for N-cadherin during cardiogenesis, and therefore rescue the lethality of the N-cadherin ablated embryos [5]. This suggests that cadherin-mediated cell-cell adhesion, rather than cadherin-specific signaling, is important during early cardiac development.

Similarly, our results also suggest that cadherin-mediated adhesion is more important than cadherin-specific signaling during the secretory stage of enamel development. We showed that two adherens junction signaling molecules (β-catenin, p120) were expressed at similar levels in WT and N-cadherin cKO enamel organs. The elimination of the E- to N-cadherin switch in secretory stage ameloblasts did not affect enamel formation and the total amount of cadherins was similar in WT and cKO sections. Therefore, cadherin-specific signaling was likely not an essential factor in enamel development, but the total cadherin-mediated cell-cell adhesion likely was important. In support of this interpretation, we have previously demonstrated that ablation of p120, which stabilizes cadherins to the cell membrane, causes severe enamel malformation [2]. In addition, we identified a potential mechanism by which E-cadherin expression is sustained in N-cadherin cKO enamel organ during the secretory stage of development. BMP2 and its antagonist NOG may play important roles in this process by up-regulating or maintaining high level E-cadherin expression beyond the pre-secretory stage and into the secretory stage of enamel development.

Our qPCR results showed that N-cadherin expression was not completely eliminated in the cKO enamel organ. Mosaic ablation of N-cadherin could account for this. However, immunohistochemical results showed little to no N-cadherin expression in the ablated ameloblasts. Perhaps, N-cadherin expression from the pulp organ that was not completely separated from the enamel organ during extraction, may have contributed to the observed N-cadherin expression in the ablated enamel organs. qPCR results demonstrated that E-cadherin expression was only about 1.5 fold greater than normal in the N-cadherin cKO enamel organ. However, since the enamel organ is an epithelial tissue, the entire enamel organ expresses E-cadherin. So, a vastly increased E-cadherin expression in just the ameloblast layer, as was observed by the immunohistochemical results, would represent a large increase in expression by the ameloblasts, but an overall lower increase in expression of total E-cadherin for the entire enamel organ. Ameloblasts compose a single cell layer that covers the forming enamel and they are extremely difficult to dissect from the enamel organ, which is why we could not extract mRNA from just the ameloblasts.

In conclusion, we demonstrated that in the absence of N-cadherin, endogenous E-cadherin expression remains at high levels in secretory stage ameloblasts. This likely compensates for the lack of N-cadherin because N-cadherin cKO mice form perfectly normal enamel. This N- to E-cadherin substitution in cKO mice is likely essential because ameloblast cadherin cell-cell attachments are essential for enamel formation [2]. Since E-cadherin can substitute for N-cadherin, cadherin-specific cell signaling is likely less important in the secretory stage enamel organ than are cell-cell attachments. Therefore, E-cadherin can functionally compensate for N-cadherin during enamel formation and the persistence of E-cadherin expression throughout the secretory stage in the N-cadherin cKO mice may be mediated by BMP2 signaling.
